# Taxing Medical Men

**Published:** 1878-11-20

**Authors:** 


### TAXING MEDICAL MEN.

In most of the States there is imp[illegible]ed a specific tax upon physicians. The last meeting of the Medical Association of Georgia, appointed a committee to m[illegible]morialize the Legislature of the State to remove the special tax of $10, now imposed upon physicians in Georgia. There are a number of medical men who are Representatives in the L[illegible]gislature, and we hope they will not shrink from u[illegible]ing the[illegible]r influence for a passage of a bill repealing the law which imposes a special tax upon physicians. No class of men of any occupation do a tit[illegible]e of the gratuitous work for the poor which is done by physicians. In all kinds of weather; in the dark hours of night when others are asleep, t[illegible]e medical practitioner, broken of his rest, and often worn down with care and anxiety, passes from one scene of distress to another, giving his time, labor and medicine to the indigent sick. It is the height of selfishness and meanness to say that he has voluntarily placed himself in such a position and shou'd therefore not complain; or that he could refuse to do this charity work. The dictates of humanity, not less than the exactions of public sentiment, leaves him no alternative.

It is clearly the duty of the State, and not of a particular class to provide for the necessities of the indigent sick. A law should be passed authorizing the authorities of each county to provide drugs, at least, if not compensation to medical men for these services and gratuities. And if this is not to be done, surely it is a small boon to ask that the specific tax upon medical men be remov-

